# Impact of acute silent ischemic lesions on clinical outcomes of carotid revascularization

**DOI:** 10.1097/JS9.0000000000000925

**Published:** 2023-12-05

**Authors:** Jie Wang, Tao Wang, Bin Yang, Yanfei Chen, Peng Gao, Yabin Wang, Jian Chen, Fei Chen, Jichang Luo, Renjie Yang, Xiaoli Min, Yan Ma, Liqun Jiao

**Affiliations:** a Department of Neurosurgery, Xuanwu Hospital, Capital Medical University; b Department of Neurology; c Department of Interventional Neuroradiology, Xuanwu Hospital, Capital Medical University, Beijing; d Department of Neurosurgery, Jinan Hospital of Xuanwu Hospital, Shandong First Medical University, Jinan; e Department of Cerebrovascular Diseases, The Second Affiliated Hospital, Kunming Medical University, Kunming, Peoples Republic of China

**Keywords:** acute silent ischemic lesion, carotid revascularization, MRI, symptomatic carotid stenosis

## Abstract

**Background:**

Previous literature has established an association between acute silent ischemic lesions (ASILs) and elevated susceptibility to future adverse clinical outcomes. The present study endeavors to scrutinize the prognostic significance of preprocedural ASILs, as detected through diffusion-weighted imaging and apparent diffusion coefficient metrics, in relation to subsequent adverse events—namely, stroke, myocardial infarction, and all-cause death—following carotid revascularization in a cohort of patients with symptomatic carotid stenosis.

**Materials and methods:**

Subjects were extracted from a comprehensive retrospective dataset involving symptomatic carotid stenosis cases that underwent carotid revascularization at a tertiary healthcare institution in China, spanning January 2019 to March 2022. Of the 2663 initially screened patients (symptomatic carotid stenosis=1600; asymptomatic carotid stenosis=1063), a total of 1172 individuals with symptomatic carotid stenosis were retained for subsequent analysis. Stratification was implemented based on the presence or absence of ASILs. The primary endpoint constituted a composite measure of in-hospital stroke, myocardial infarction, or all-cause death. Both carotid endarterectomy (CEA) and carotid artery stenting (CAS) treatment modalities were individually subjected to propensity score-matched analyses.

**Results:**

Among the 584 subjects who underwent CEA, 91 ASIL-positive and 91 ASIL-negative (NASIL) cases were propensity score-matched. Notably, the ASIL cohort demonstrated a statistically significant augmentation in the risk of primary outcomes relative to the NASIL group [10.99 vs. 1.10%; absolute risk difference, 9.89% (95% CI: 3.12–16.66%); RR, 10.00 (95% CI: 1.31–76.52); *P*=0.01]. Similarly, within the 588 CAS-treated patients, 107 ASIL-positive and 107 NASIL cases were matched, revealing a correspondingly elevated risk of primary outcomes in the ASIL group [9.35 vs. 1.87%; absolute risk difference, 7.48% (95% CI: 1.39–13.56%); RR, 5.00 (95% CI: 1.12–22.28); *P*=0.02].

**Conclusions:**

ASILs portend an elevated risk for grave adverse events postcarotid revascularization, irrespective of the specific revascularization technique employed—be it CEA or CAS. Thus, ASILs may serve as a potent biomarker for procedural risk stratification in the context of carotid revascularization.

## Introduction

Highlights

- The preprocedural acute silent ischemic lesions identified by diffusion-weighted imaging and apparent diffusion coefficient were related to a high-risk of serious adverse events in patients who receiving carotid revascularization.
- The acute silent ischemic lesion was a potential biomarker for procedural risk and those with acute ischemic lesions need more attention considering carotid revascularization.

Silent ischemic lesions represent cerebral infarcts devoid of concurrent stroke-like symptomatic manifestations, a concept corroborated by existing literature^1,2^. Prevalence rates of these silent cerebral infarcts fluctuate between 8 and 28%, and manifest an escalation concomitant with advancing age^3–5^. In patients diagnosed with carotid artery stenosis, a recurrent emergence of ischemic lesions has been discerned in MRI subsequent to the index ischemic event. Morphological attributes delineated in MRI sequences serve as reliable indices for lesion staging^6,7^. Acute ischemic lesions (AILs) are optimally visualized through diffusion-weighted imaging (DWI) and apparent diffusion coefficient (ADC) parameters on MRI^6–8^. A heightened DWI signal coupled with a reduced ADC signal serves as an indicator of an AIL that transpired within a temporal bracket of 10 days, with sensitivity and specificity rates poised at 88 and 90%, respectively^9^. Consequently, acute silent ischemic lesions (ASILs) are defined as those satisfying the above-mentioned MRI criteria without exhibiting correlating symptoms within a decadal day framework prior to MRI examination. ASILs are substantiated to be concomitant with an elevated risk of prospective adverse incidents, encompassing future ischemic events as well as cognitive deterioration^1,4,5,10–12^.

In 2004, Rothwell *et al*. executed an individual patient meta-analysis integrating data from the European Carotid Surgery Trial (ECST) and the North American Symptomatic Carotid Endarterectomy Trial (NASCET). Their findings illuminated that the efficacy of carotid revascularization wanes considerably when elongating the latency between symptom onset and surgical intervention^13–15^. For individuals presenting with carotid stenosis of 50% or greater, the number needed to treat to avert a single ipsilateral stroke over a 5-year horizon was five for subjects randomized within a fortnight subsequent to their most recent ischemic incident, in contrast to 125 for those randomized beyond a 12-week threshold^13^. The most contemporary guidelines issued by the European Stroke Organization (ESO) recommend early carotid endarterectomy (CEA) in symptomatic patients manifesting 50–99% carotid stenosis, ideally within 14 days following the initial ischemic index event^16^. Predicated upon these clinical guidelines, an increasing proportion of patients are undergoing expeditious CEA over the preceding two decades. For instance, a study in the United States have substantiated that the median latency between transient ischemic attack (TIA)/stroke incidence and CEA has diminished from 22 days in 2009 (IQR 10–56) to 12 days in 2014 (IQR 7–26), with the share of patients receiving therapy within a 14-day interval burgeoning from 37 to 58%^17^. Similarly, German data spanning from 2003 to 2014 revealed a contraction in the interval from ischemic index events to CEA, decreasing from 28 to 8 days^18^. Nonetheless, in China, the proportion of patients who undergo carotid revascularization within the designated 14-day window remains relatively attenuated, ostensibly due to apprehensions concerning elevated peri-procedural complications in the acute phase.

The incidence of ASILs is notably elevated in patients diagnosed with symptomatic carotid stenosis who are impending carotid revascularization, particularly in those surpassing the prespecified early revascularization interval of 14 days. Given the asymptomatic nature of preprocedural silent ischemic lesions, such occurrences frequently escape clinical attention in patients slated for carotid revascularization. Within the ambit of clinical practice, it has been empirically observed that patients manifesting novel infarcts on preprocedural MRI are disproportionately susceptible to an increased incidence of postprocedural adverse events. Despite these preliminary observations, the ramifications of ASILs on the clinical outcomes of carotid revascularization in patients whose interval from the most recent ischemic event to the revascularization procedure exceeds 14 days remain inadequately elucidated. Our aim was to evaluate impact of preprocedural ASIL identified by DWI and ADC on adverse clinical events after carotid revascularization in patients who outstripped the early revascularization time (14 days) based on propensity-matched analysis of a large retrospective cohort.

## Methods

### Dataset

The current investigation rigorously conformed to the Strengthening the Reporting of Cohort, Cross-Sectional, and Case–Control Studies in Surgery 2021 (STROCSS 2021, Supplemental Digital Content 1, http://links.lww.com/JS9/B513) guidelines, as delineated for observational research endeavors^19^. This inquiry was registered in a retrospective manner on the ResearchRegistry platform. Ethical approval was procured from the Institutional Ethics Committee of a tertiary medical center situated in the People’s Republic of China, under the designation [2022]113. Given the retrospective study design, the prerequisite for informed consent was judiciously abrogated. Data germane to the study’s objectives were obtained from digital medical archives. Any personally identifiable information such as names, addresses, and social security numbers was stripped from the collected data and replaced of personal identifiers with a unique code, making it impossible to link the data to specific individuals.

### Patients

Individuals manifesting symptomatic atherosclerotic carotid stenosis, localized either at the carotid bifurcation or within the internal carotid artery, and who were subjected to CAS and CEA, were selected through a retrospective and continuous screening process spanning January 2019 to March 2022, facilitated at a tertiary medical institution in China. Inclusion parameters comprised: 1) a definitive diagnosis of symptomatic carotid stenosis; 2) an interval exceeding 14 days between the most recent ischemic episode and the carotid revascularization procedure; and 3) administration of MRI within a 48 h window antecedent to carotid revascularization. Exclusion criteria:1) cases of nonatherosclerotic carotid stenosis; 2) individuals undergoing stenting for intracranial carotid lesions; 3) patients subjected to CEA in concurrence with hybrid surgical interventions entailing proximal or distal arterial stenting; and 4) a time lapse of fewer than 14 days between the latest ischemic event and carotid revascularization. Patients manifesting abrupt neurological symptomatology within the anatomical purview of the carotid artery stenosis during the preceding 6 months were categorized as symptomatic. The term ‘ischemic event’ encapsulates both TIA and ischemic stroke, while ‘carotid revascularization’ refers to either CEA or CAS.

Patients were partitioned into two discrete cohorts: the Acute Silent Ischemic Lesion (ASIL) group and the Non-Acute Silent Ischemic Lesion (NASIL) group. The criterion for ASIL classification involved: 1) elevated signal intensity on DWI coupled with diminished signal on ADC; and 2) absence of novel symptomatology within a 10-day period anteceding the MRI evaluation. All pertinent imaging was subjected to meticulous scrutiny by the IsCore Image Core Laboratory (http://imagecorelabcn.com/en/), which remained explicitly disengaged from statistical computations and was blinded to patients’ clinical data.

### Carotid revascularization protocol

The therapeutic regimen for carotid revascularization was meticulously aligned with the guidelines promulgated by the American Heart Association/American Stroke Association (AHA/ASA)^20^. For patients who presented with a recent TIA or ischemic stroke, coupled with ipsilateral moderate-to-severe carotid stenosis (ranging from 50 to 99%), as corroborated via catheter-based or noninvasive imaging modalities, CEA or CAS was strongly advocated to mitigate the prospective incidence of subsequent cerebrovascular events^20^. Surgeons engaged in this study demonstrated proficiency in independently executing both CEA and CAS procedures. In the current investigative context, CEA was preferentially designated as the first-line therapeutic strategy, customarily administered under general anesthesia. CAS was carried out under local anesthesia in scenarios where patients were evaluated as manifesting elevated risk factors for CEA. Risk factors for CEA were appraised from dual perspectives, encompassing intolerance to general anesthesia and inappropriateness of surgical techniques. Salient contra-indicatory elements for CEA primarily consisted of nonpatent communicating arteries and technical challenges arising from elevated carotid artery bifurcation. The choice of stent type, as well as the specific technique and devices employed, were left to the discretionary judgment of the treating physician. Moreover, patient and familial preferences were accorded consideration in instances devoid of unequivocal indications or contraindications for either CEA or CAS.

Patients were administered monotherapy with antiplatelet agents—comprising aspirin (100 mg daily), clopidogrel (75 mg daily), or ticagrelor (90 mg twice daily)—for a minimum duration of 5 days antecedent to CEA. Alternatively, dual antiplatelet therapy—entailing the combination of aspirin (100 mg daily) and either clopidogrel (75 mg daily) or ticagrelor (90 mg twice daily)—was instituted for at least 5 days prior to CAS. In instances where patients had not received the prescribed 5-day antiplatelet regimen, a loading dose of supplemental antiplatelet medication was administered preoperatively. Subsequent to the revascularization procedures, the continuance of mono or dual antiplatelet therapy was sustained, barring the presence of contraindications. Risk factor management strategies were consistently applied in accordance with AHA/ASA guidelines^20^.

### Outcomes

The primary outcomes was a composite of in-hospital any stroke, myocardial infarction (MI) or all-cause death. Secondary outcomes were delineated as follows: 1) incidence of any stroke, 2) ischemic stroke, 3) hemorrhagic stroke, 4) all-cause death, 5) MI, 6) procedure related complications, and 7) postoperative ASILs. ‘Any stroke’ was characterized by the acute manifestation of clinical indicators indicative of localized or global cerebral functional disturbances, corroborated by evidence of novel hemorrhagic or ischemic lesions on postoperative imaging modalities, such as MRI or computed tomography (CT). MI was defined on the basis of one or multiple criteria, including electrocardiographic alterations suggestive of acute MI and a troponin elevation surpassing thrice the upper reference limit within the milieu of suspected myocardial ischemia. Complications relevant to the procedure encompassed pulmonary embolism, reoperative interventions, cerebral hyperperfusion syndrome, neck hematoma in the context of CEA, and access site hematoma for CAS. Parameters for pulmonary embolism included acute dyspnea or exacerbated existing dyspnea, chest discomfort, syncope or dizziness due to hypotension or shock, hemoptysis, tachycardia, or tachypnea, substantiated by abnormal findings on chest radiography, electrocardiography, lower extremity venous ultrasonography, or arterial blood gas analysis^21^.

### Statistical analysis

Continuous variables were articulated as mean values with accompanying SD or as medians with associated ranges. Categorical variables were reported as enumerations and corresponding percentages. The cardinal predictive variable selected for the analytical evaluation was the presence of new infarctions on preprocedural MRI, specifically differentiating between the ASIL group and the NASIL group. To enhance the internal validity and comparability between these demarcated groups, a propensity score matching (PSM) methodology was employed. Segregated analyses were conducted for patients subjected to CEA and CAS, following the application of propensity scores derived from logistic regression models. The incorporated variables within these models included age, sex, hypertension, diabetes mellitus, coronary artery disease, peripheral artery disease, lipid disorders, atrial fibrillation, BMI, smoking history, alcohol consumption history, degree of stenosis in the symptomatic qualifying artery, and time from latest ischemic event to revascularization. Subsequent to this, a one-to-one propensity score-matched analysis was conducted, isolating patients with available data within the ASIL and NASIL categories. Calibration of treatment cohorts was achieved utilizing these propensity scores, with an absolute unit calibration of 0.25. Covariate equilibrium between the cohorts, both pre-PSM and post-PSM, was evaluated through standardized differences; imbalances of significance were categorized by a standardized difference exceeding 0.2.

For each outcome and comparative assessment, unadjusted and propensity score-matched event numbers, incidence rates, standardized differences, and relative risks were calculated, each with 95% CIs. In-hospital outcomes amongst the matched groups were subjected to χ^2^ testing. A *P*-value less than 0.05 was deemed to denote statistical significance, and all statistical tests were bifacially conducted. The computational analysis was executed employing SAS software, version 9.4 (SAS Institute).

## Result

**Figure 1:**
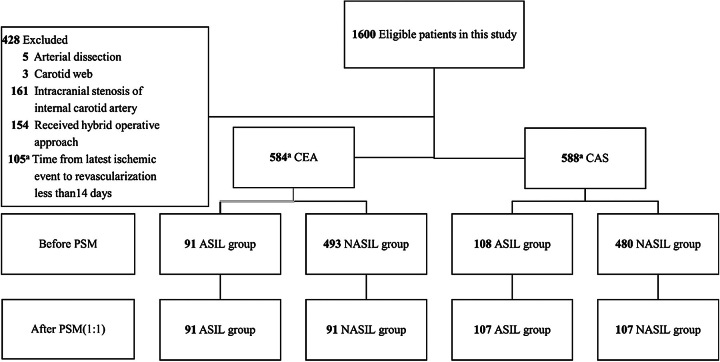
Flowchart of patient selection a These 1277 patients were divided into 4 groups according to whether they had carotid revascularization within 14 days after latest ischemic event occurrence and whether there were acute ischemic lesions on pre-procedural MRI. Then, the incidence of in hospital stroke, myocardial infarction, and death in these 4 groups were statistically analyzed in Figure 2 and Figure 3.

Within the specified timeframe extending from January 2019 to March 2022, an aggregate of 2663 patients were scrupulously assessed for eligibility, having undergone carotid revascularization procedures (symptomatic carotid stenosis=1600; asymptomatic carotid stenosis=1063). Out of these, 1172 symptomatic patients were duly incorporated into the analytic evaluation. 584 patients with CEA [mean (SD) age, 63.49 (7.81) years; 501 (85.79%) male] and 588 with CAS [mean (SD) age, 66.62 (7.87) years; 507 (86.22%) male] (Fig. 1).

As a means of assessing the global surgical proficiency of this medical institution, we conducted an exhaustive investigation into the perioperative complication rates among all symptomatic patients subjected to CEA or CAS. We collected all adverse events between postoperative and discharge. The average length of stay in hospital was 3 days. The aggregated rate of complications—encompassing stroke, MI, or all-cause death—was found to be 3.47% for CEA and 4.51% for CAS procedures.

**Figure 2:**
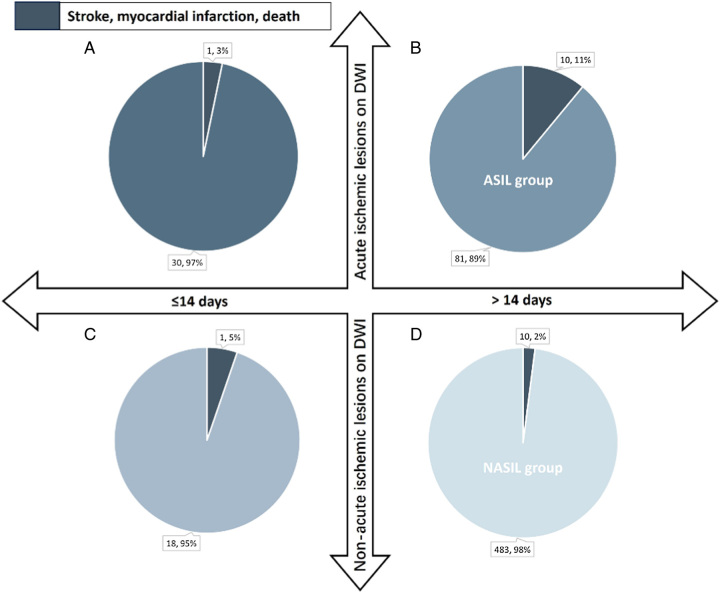
In-hospital stroke, myocardial infarction or death after carotid endarterectomy in symptomatic patients. Patients were divided into four groups that according to whether they had carotid revascularization within 14 days after latest ischemic event occurrence and whether there were acute ischemic lesions on preprocedural MRI. a) carotid revascularization within 14 days after latest ischemic event occurrence and there were acute ischemic lesions on preprocedural MRI b) carotid revascularization more than 14 days after latest ischemic event occurrence and there were acute ischemic lesions on preprocedural MRI c) carotid revascularization within 14 days after latest ischemic event occurrence and there were nonacute ischemic lesions on preprocedural MRI d) carotid revascularization more than 14 days after latest ischemic event occurrence and there were nonacute ischemic lesions on preprocedural MRI.

**Figure 3:**
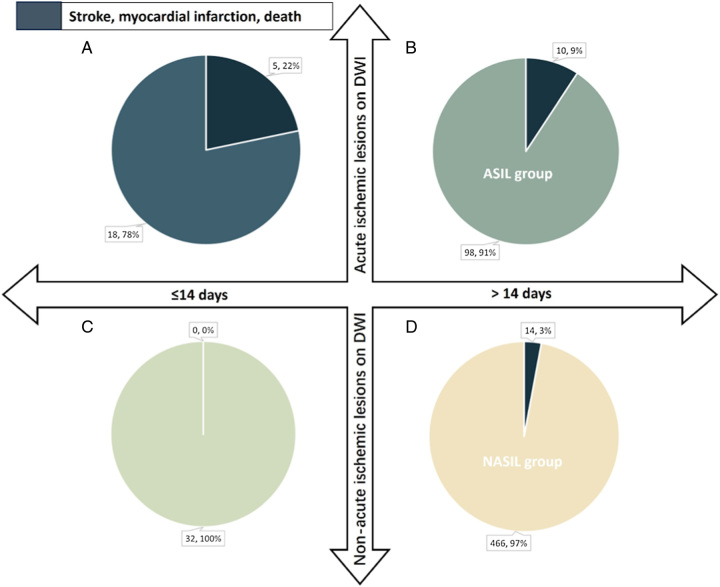
In-hospital stroke, myocardial infarction or death after carotid stenting in symptomatic patients. Patients were divided into four groups that according to whether they had carotid revascularization within 14 days after latest ischemic event occurrence and whether there were acute ischemic lesions on preprocedural MRI. a) carotid revascularization within 14 days after latest ischemic event occurrence and there were acute ischemic lesions on preprocedural MRI b) carotid revascularization more than 14 days after latest ischemic event occurrence and there were acute ischemic lesions on preprocedural MRI c) carotid revascularization within 14 days after latest ischemic event occurrence and there were nonacute ischemic lesions on preprocedural MRI d) carotid revascularization more than 14 days after latest ischemic event occurrence and there were non-acute ischemic lesions on preprocedural MRI.

Concurrently, 1277 patients diagnosed with symptomatic carotid artery stenosis were categorically subdivided into four distinct cohorts. This stratification was predicated on two variables: 1) whether carotid revascularization was executed within 14 days subsequent to the most recent ischemic event, and 2) the presence or absence of AILs on preoperative MRI. Thereafter, a statistical analysis was executed, focusing on the incidence rates of in-hospital stroke, MI, and death across these four stratified groups (refer to Fig. 2 and Fig. 3 for details).

For patients who received CEA, 91 in the ASIL group [mean (SD) age, 63.14 (8.29) years; 80 (87.91%) male] and 91 in the NASIL group [mean (SD) age, 62.98 (7.24) years; 80 (87.91%) male] were matched with same propensity score. The time from latest ischemic event to revascularization were 44 (IQR 38) days in the ASIL group and 58 days (IQR 35) in the NASIL group. 96.7% patients were severe stenosis either the ASIL group or the NASIL group. For patients who received CAS, 107 in the ASIL group [mean (SD) age, 67.43 (8.06) years; 89 (83.18%) male] and 107 in the NASIL group [mean (SD) age, 67.93 (7.78) years; 89 (83.18%) male] were matched similarly (Table 1). The time from latest ischemic event to revascularization were 40 (IQR 35) days in the ASIL group and 43 days (IQR 34) in the NASIL group. 92.52% patients in the ASIL group and 95.3% patients in the NASIL group were severe stenosis.

**Table 1:** Baseline characteristics of patients before and after propensity-score matching (1:1).

	Carotid endarterectomy No. (%)						Carotid artery stenting No. (%)					
	All patients (*n*=584)			Propensity score-matched patients (*n*=182)			All patients (*n*=588)			Propensity score-matched patients (*n*=214)		
	ASIL (*n*=91)	NASIL (*n*=493)	Standardized difference^a^	ASIL (*n*=91)	NASIL (*n*=91)	Standardized difference^a^	ASIL (*n*=108)	NASIL (*n*=480)	Standardized difference^a^	ASIL (*n*=107)	NASIL (*n*=107)	Standardized difference^a^
Age, mean (SD), years	63.14 (8.29)	63.56 (7.73)	−0.07	63.14 (8.29)	62.98 (7.24)	0.02	67.36 (8.06)	66.45 (7.83)	0.05	67.43 (8.06)	67.93 (7.78)	0.10
Distribution, years												
≤60	32 (35.16)	150 (30.43)	0.10	32 (35.16)	29 (31.87)	0.07	23 (21.30)	105 (21.88)	−0.01	22 (20.56)	19 (17.76)	0.07
60–70	44 (48.35)	265 (53.75)	−0.11	44 (48.35)	51 (56.04)	−0.15	49 (45.37)	235 (48.96)	−0.07	49 (45.79)	48 (44.86)	0.02
70–80	15 (16.48)	73 (14.81)	0.05	15 (16.48)	11 (12.09)	0.12	32 (29.63)	119 (24.79)	0.11	32 (29.91)	34 (31.78)	−0.04
＞80	0	5 (1.01)	−0.14	0	0	0.00	4 (3.70)	21 (4.38)	−0.03	4 (3.74)	6 (5.61)	−0.09
Sex												
Male	80 (87.91)	421 (85.40)	0.07	80 (87.91)	80 (87.91)	0.00	90 (83.33)	417 (86.88)	−0.10	89 (83.18)	89 (83.18)	0.00
Female	11 (12.09)	72 (14.60)	−0.07	11 (12.09)	11 (12.09)	0.00	18 (16.67)	63 (13.13)	0.10	18 (16.82)	18 (16.82)	0.00
Hypertension	67 (73.63)	361 (73.23)	−0.01	67 (73.63)	72 (79.12)	0.12	82 (75.93)	359 (74.79)	−0.03	81 (75.70)	83 (77.57)	0.04
Diabetes	42 (46.15)	159 (32.25)	−0.28	42 (46.15)	45 (49.45)	0.07	56 (51.85)	173 (36.04)	−0.32	55 (51.40)	59 (55.14)	0.07
Coronary artery disease	19 (20.88)	73 (14.81)	−0.16	19 (20.88)	23 (25.27)	0.10	18 (16.67)	110 (22.92)	0.16	18 (16.82)	15 (14.02)	−0.07
Peripheral artery disease	1 (1.10)	7 (1.42)	0.03	1 (1.10)	2 (2.20)	0.09	4 (3.70)	10 (2.08)	−0.10	3 (2.80)	1 (0.93)	−0.11
Lipid disorder	41 (45.05)	206 (41.78)	−0.07	41 (45.05)	49 (53.85)	0.17	46 (42.59)	208 (43.33)	0.01	45 (42.06)	36 (33.64)	−0.17
Atrial fibrillation	1 (1.10)	7 (1.42)	0.03	1 (1.10)	0	−0.09	3 (2.78)	9 (1.88)	−0.06	3 (2.80)	3 (2.80)	0.00
BMI												
<28	76 (83.52)	413 (83.77)	−0.01	76 (83.52)	79 (86.81)	−0.09	93 (86.11)	395 (82.29)	0.10	92 (85.98)	93 (86.92)	−0.03
≥28	15 (16.48)	80 (16.23)	0.01	15 (16.48)	12 (13.19)	0.09	15 (13.89)	85 (17.71)	−0.10	15 (14.02)	14 (13.08)	0.03
Smoking history	60 (65.93)	287 (58.22)	−0.16	60 (65.93)	63 (69.23)	0.07	50 (46.30)	305 (63.54)	0.35	50 (46.73)	51 (47.66)	0.02
Alcohol history	46 (50.55)	218 (44.22)	−0.13	46 (50.55)	45 (49.45)	−0.02	43 (39.81)	218 (45.42)	0.11	42 (39.25)	43 (40.19)	0.02
Stenosis of symptomatic qualifying artery												
% Stenosis, median (IQR)	85.48 (5.75)	84.78 (8.00)	−0.08	85.48 (5.75)	85.10 (7.92)	0.00	82.58 (8.19)	82.14 (8.25)	−0.06	82.61 (8.41)	82.56 (7.72)	0.10
Distribution, % stenosis												
50–69	3 (3.30)	24 (4.87)	−0.08	3 (3.30)	3 (3.30)	0.00	8 (7.41)	44 (9.17)	−0.06	8 (7.48)	5 (4.67)	0.10
70–99	88 (96.70)	469 (95.13)	0.08	88 (96.70)	88 (96.70)	0.00	100 (92.59)	436 (90.83)	0.06	99 (92.52)	102 (95.33)	−0.10
Time from latest ischemic event to revascularization, median (IQR), days	40 (38.00)	66 (88.00)	−0.57	40 (38.00)	58 (35.00)	−0.06	39 (35.00)	61 (61.00)	−0.38	40 (35.00)	43 (34.00)	−0.10

a Standardized difference of >0.2 indicates significant difference.

ASIL, acute silent ischemic lesions; NASIL, non-acute silent ischemic lesions.

Baseline characteristics and concomitant medical conditions, both pre-PSM and post-PSM, have been tabulated comprehensively in Table 1. Amongst these propensity score-matched pairs, pertinent data pertaining to in-hospital adverse clinical events were available for exhaustive analysis for the entire patient population.

### Primary outcome

In the cohort of patients subjected to CEA, the primary composite outcome, which encompasses the in-hospital incidence of stroke, MI, or death, manifested as 10.99% in the ASIL group compared to a mere 1.10% in the NASIL group. The absolute difference between these groups was statistically significant, at 9.89% [95% CI, 3.12% to 16.66%; Relative Risk (RR), 10.00 (95% CI: 1.31–76.52); *P*=0.01] (refer to Table 2 for comprehensive data).

**Table 2:** In-hospital perioperative outcomes after propensity-score matching in carotid endarterectomy.

Outcomes	ASIL (*N*=91)	NASIL (*N*=91)	Absolute difference (95% CI), %	Relative risk (95% CI)	*P*
Primary outcomes					
Any stroke, myocardial infarction or all-cause death	10 (10.99)	1 (1.10)	9.89 (3.12–16.66)	10.00 (1.31–76.52)	0.01
Secondary outcomes					
Any stroke	8 (8.79)	1 (1.10)	7.69 (1.49–13.89)	8.00 (1.02–62.67)	0.04^a^
Ischemic stroke	6 (6.59)	0	NA	NA	NA
Hemorrhagic stroke	2 (2.20)	1 (1.10)	1.10 (−2.60–4.80)	2.00 (0.18–21.67)	1.00^a^
All-cause death	2 (2.20)^b^	1 (1.10)^c^	1.10 (−2.60–4.80)	2.00 (0.18–21.67)	1.00^a^
Myocardial infarction	2 (2.20)	0	NA	NA	NA
Procedure related complications	6 (6.59)	2 (2.20)	4.40 (−1.53–10.32)	3.00 (0.62–14.47)	0.28^a^
Pulmonary embolism	0	0	NA	NA	NA
Re-operation	1 (1.10)^d^	0	NA	NA	NA
Cerebral hyperperfusion syndrome	5 (5.49)	1 (1.10)	4.40 (−0.75–9.54)	5.00 (0.60–41.96)	0.21^a^
Neck hematoma	0	1 (1.10)	NA	NA	NA
Postoperative acute silent ischemic lesions	26 (28.57)	19 (20.88)	7.69 (−4.79–20.18)	1.37 (0.81–2.29)	0.23

a The cells have expected counts less than 5.

χ^2^ may not be a valid test. Continuity Adj. χ^2^ was performed.

b Both patients died of cardiac arrest due to postoperative acute myocardial infarction.

c The patient died of cerebral hernia induced by intracranial hypertension due to postoperative cerebral hemorrhage.

d Acute ipsilateral carotid artery occlusion after carotid endarterectomy, emergency thrombectomy was performed.

ASIL, acute silent ischemic lesions; NASIL, non-acute silent ischemic lesions.

In a similar vein, for patients who underwent CAS, the primary outcome demonstrated 9.35% in the ASIL group as opposed to 1.87% in the NASIL group. The absolute discrepancy was 7.48% [95% CI: 1.39–13.56%; RR, 5.00 (95% CI: 1.12–22.28); *P*=0.02] (please consult Table 3 for detailed elucidation).

**Table 3:** In-hospital perioperative outcomes after propensity-score matching in carotid artery stenting.

Outcomes	ASIL (*N*=107)	NASIL (*N*=107)	Absolute difference (95% CI), %	Relative risk (95% CI)	*P*
Primary outcomes					
Any stroke, myocardial infarction or all-cause death	10 (9.35)	2 (1.87)	7.48 (1.39–13.56)	5.00 (1.12–22.28)	0.02
Secondary outcomes					
Any stroke	10 (9.35)	2 (1.87)	7.48 (1.39–13.56)	5.00 (1.12–22.28)	0.02
Ischemic stroke	7 (6.54)	1 (0.93)	5.61 (0.58–10.63)	7.00 (0.88–55.92)	0.07^a^
Hemorrhagic stroke	3 (2.80)	1 (0.93)	1.87 (−1.75–5.49)	3.00 (0.32–28.39)	0.61^a^
All-cause death	1 (0.93)^b^	0	NA	NA	NA
Myocardial infarction	0	0	NA	NA	NA
Procedure related complications	7 (6.54)	2 (1.87)	4.67 (−0.67–10.01)	3.50 (0.74–16.46)	0.17^a^
Pulmonary embolism	1 (0.93)	0	NA	NA	NA
Re-operation	0	0	NA	NA	NA
Cerebral hyperperfusion syndrome	6 (5.61)	1 (0.93)	4.67 (−0.05–9.40)	6.00 (0.73–49.00)	0.12^a^
Access site bleeding complication	0	1 (0.93)	NA	NA	NA
Postoperative acute silent ischemic lesions	63 (48.84)	66 (51.16)	−2.80 (−15.91−10.30)	0.95 (0.77−1.19)	0.67

a The cells have expected counts less than 5. χ^2^ may not be a valid test. Continuity Adj. χ^2^ was performed.

b The patient died of cerebral hernia induced by intracranial hypertension due to postoperative cerebral hemorrhage.

ASIL, acute silent ischemic lesions; NASIL, non-acute silent ischemic lesions.

### Secondary outcomes

In the domain of secondary outcomes, patients who received CEA and were part of the ASIL group manifested significantly elevated risks in both stroke [8.79 versus 1.10%; absolute difference, 7.69% (95% CI: 1.49–13.89%); RR, 8.00 (95% CI: 1.02–62.67); *P*=0.04] and ischemic stroke (6.59 versus 0%). No statistically significant differences were observed between the two groups for outcomes such as hemorrhagic stroke, death, procedure-associated complications, and postoperative ASILs (refer to Table 2 for granular data).

Likewise, in the CAS cohort, the ASIL group also exhibited markedly augmented risks in both stroke [9.35 versus 1.87%; absolute difference, 7.48% (95% CI: 1.39–13.56%); RR, 5.00 (95% CI: 1.12–22.28); *P*=0.02] and ischemic stroke [6.54 versus 0.93%; absolute difference, 5.61% (95% CI: 0.58–10.63%); RR, 7.00 (95% CI: 0.88–55.92); *P*=0.07]. Analogous to the CEA cohort, the outcomes did not diverge statistically in terms of hemorrhagic stroke, death, procedure-associated complications, and postoperative ASILs (refer to Table 3 for an exhaustive account).

Prior to the application of PSM, the analytical outcome delineating the ASIL group vis-à-vis the NASIL group can be accessed in Supplementary Tables S1 (Supplemental Digital Content 2, http://links.lww.com/JS9/B514) and S2 (Supplemental Digital Content 2, http://links.lww.com/JS9/B514). Regardless of whether CEA or CAS was performed, the ASIL group invariably demonstrated a higher cumulative incidence of primary composite outcomes [CEA: 10.99 versus 2.03%; absolute difference, 8.96% (95% CI: 2.42–15.51%); RR, 5.42 (95% CI: 2.32–12.64); *P*<0.001]. [CAS: 9.26 versus 2.92%; absolute difference, 6.34% (95% CI: 0.67–12.01%); RR, 3.17 (95% CI: 1.45–6.95); *P*=0.01].

### CEA versus CAS

For the same population, in ASIL group, there was no significant difference in primary outcomes between CAS and CEA [10.99 versus 9.26%; absolute difference, 1.73% (95% CI: −6.71–10.17%); RR, 1.01 (95% CI: 0.93–1.12); *P*=0.69] (see Supplementary Material Table S3, Supplemental Digital Content 2, http://links.lww.com/JS9/B514).

In NASIL group, there was also no significant difference in primary outcomes between CAS and CEA [2.03 versus 2.92%; absolute difference, −0.89% (95% CI: −2.84–1.06%); RR, 0.99 (95% CI: 0.97–1.01); *P*=0.37] (see Supplementary Material Table S4, Supplemental Digital Content 2, http://links.lww.com/JS9/B514).

## Discussion

### Stroke, MI, and death

The present investigation constitutes the inaugural study to systematically assess the ramifications of preprocedural ASIL, as identified via DWI and ADC, on postcarotid revascularization adverse clinical outcomes among patients who exceeded the early revascularization window (>14 days). Given the study’s pivotal focus on surgical safety, three critical adverse events—namely, stroke, MI, and all-cause death—were selected as the primary safety outcomes. Within this tertiary healthcare institution, the aggregate complication rate for patients undergoing CEA was noted to be 3.47%, while it was 4.51% for those who received CAS, specifically among symptomatic individuals. Notably, individuals exhibiting preprocedural ASIL presented with a heightened composite event rate encompassing stroke, MI, and death, both in the CEA cohort [10.99 versus 1.10%; relative risk (RR), 10.00 (95% CI: 1.31–76.52); *P*=0.01] and the CAS cohort [9.35 versus 1.87%; RR, 5.00 (95% CI: 1.12–22.28); *P*=0.02]. A review of extant literature published post-2005 revealed that for symptomatic patients undergoing carotid revascularization, the perioperative stroke/death rate for the CEA and CAS groups stood at 2.68 and 4.69%, respectively^22^. Such findings accentuate that the adverse event incidence in the ASIL cohort considerably supersedes those reported in previous literature. For this specific population, no significant divergence in outcomes was noted between CEA and CAS within the ASIL group [10.99% versus 9.26%, RR, 1.01 (95% CI: 0.93–1.12); *P*=0.69]. Hence, it is implicated that neither CEA nor CAS may be optimally suited for patients with ASIL, as both surpass the 6% perioperative complication threshold recommended by existing guidelines^23^.

### Hemorrhagic stroke and ischemic stroke

With respect to secondary outcomes, no statistically significant variances were observed in the occurrence of hemorrhagic stroke between the two cohorts [CEA: 2.20 versus 1.10%; RR, 2.00 (95% CI: 0.18–21.67); *P*=1.00 CAS: 2.80 versus 0.93%; RR, 3.00 (95% CI: 0.32–28.39); *P*=0.61]. However, a markedly elevated incidence of ischemic stroke was registered in the ASIL group compared to the NASIL group [CEA: 6.59 versus 0%; CAS: 6.54 versus 0.93%; RR, 7.00 (95% CI: 0.88–55.92); *P*=0.07]. Such data intimate that ischemic stroke largely underpins the observed disparities between the groups.

Postoperative hemorrhagic stroke is predominantly ascribed to ipsilateral cerebral hemisphere hyperperfusion, generally resultant from suboptimal perioperative blood pressure regulation^24,25^. In this research endeavor, meticulous perioperative blood pressure monitoring was implemented, ensuring conformity with, or slight reductions from, preoperative levels; thus, hyperperfusion-induced cerebral hemorrhage incidences were comparable between the ASIL and NASIL groups.

To elucidate the etiological basis of ischemic stroke, image core-lab reinterpretation of postoperative MRI was executed in patients who experienced ischemic stroke postrevascularization. The probable mechanism underlying ischemic stroke—whether artery-to-artery embolism, perforator occlusion, hypoperfusion, or a hybrid mechanism—was inferred based on infarct patterns evident in the MRIs^26,27^. Outcomes manifested that postoperative ischemic stroke was predominantly ipsilateral, with artery-to-artery embolism being the chief mechanistic factor (Supplementary Material Table S5, Supplemental Digital Content 2, http://links.lww.com/JS9/B514). These data suggest that ASIL presence on DWI may signify impaired embolic clearance and thus, could serve as an indicator of unstable plaque formation.

Prior investigations have postulated that the presence of multiple silent lesions upon admission portends an elevated susceptibility to early recurrent stroke^2,28^. Recent analyses examining 30-day and 90-day recurrence of ischemic lesions via MRI indicate a protracted vulnerability to stroke that persists up to 90 days^2^. Meanwhile, the revascularization process poses a proclivity for embolic dislodgment, culminating in subsequent embolic events. This is corroborated by the unanticipatedly high incidence of microemboli, as detected via Transcranial Doppler (TCD) ultrasonography, both spontaneously and intrarevascularization or postrevascularization^29^. Thus, the question of whether postoperative ischemic stroke is a sequel of carotid revascularization or a manifestation of natural history, and whether the intervention in individuals with ASIL mitigates or exacerbates the risk of subsequent ischemic stroke, requires substantiation through future comparative studies involving medical therapy vis-à-vis carotid revascularization.

### Significance and prospects

This study holds the distinction of being the inaugural exploration focusing on the ramifications of preprocedural ASIL on outcomes postcarotid revascularization in patients who surpassed the optimal temporal threshold for revascularization (14 days), thereby identifying a novel high-risk surgical demographic. In the context of patients presenting with symptomatic carotid stenosis ranging between 50 and 99%, ipsilateral recurrent ischemic stroke rates prerevascularization were quantified as follows: 2.7% (1 days), 5.3% (3 days), 11.5% (14 days), and 18.8% (90 days)^30^. Hence, it is elucidated that for patients who exceed the optimal time window for revascularization (>14 days), the high-risk epoch for recurrent stroke has ostensibly elapsed, and the protracted recurrence rate ~10%. Analogously, the postprocedural risk for patients manifesting ASIL approximates the same percentage. Such patients do not evidently accrue additional benefits from revascularization. The manifestation of ASIL could signify an impaired capacity for embolic clearance (washout)^27^. Under this state, the revascularization procedure may exacerbate plaque instability and instigate blood-brain barrier dysfunction. Conversely, deferring the procedure postischemic event may facilitate cerebrovascular auto-regulation and plaque stabilization, thereby circumventing some of the aforementioned adverse outcomes^31,32^. Accordingly, surgical interventions for this cohort necessitate judicious selection.

With respect to the therapeutic stratification for symptomatic carotid stenosis, the findings of extant randomized controlled trials (RCTs) have enshrined the primacy of revascularization as a first-line intervention. The future trajectory of research in this domain pertains to the precision of treatment, entailing the meticulous screening of high-risk candidates for revascularization and the subsequent re-evaluation of their surgical risks and benefits. The salient contribution of the present study resides in its deployment of readily accessible imaging modalities to isolate a high-risk patient cluster. The employed screening methodology is both convenient and extendable, conferring pivotal clinical implications. So, we recommend routine administration of MRI within a 48 h window antecedent to carotid revascularization.

### Limitations

Nevertheless, the limitations of the current study are nontrivial. In the matching of propensity scores, no indicators were included to evaluate the severity of ischemic events. Consequently, the study may be confounded by the difference in severity and extent of patients included in the study. Moreover, the study would benefit from the inclusion of infarct location and volume for stratified analysis. Another shortfall is the confinement to in-hospital adverse clinical events, which precludes insights into the long-term prognostic relevance of these findings. As such, extended follow-up of the ASIL cohort who underwent carotid revascularization is imperative for a nuanced understanding of both clinical and subclinical events in this patient demographic.

## Conclusion

For symptomatic patients with carotid artery stenosis, ASILs identified by DWI and ADC were related to a high-risk of postprocedural serious adverse events, especially ischemic events, for carotid revascularization. The acute silent ischemic lesion was a potential biomarker for procedural risk and those with AILs need more attention considering carotid revascularization, which warrants future prospective studies.

## Ethical approval

The study procedures were approved by Ethics Committee of Xuanwu Hospital, Capital Medical University No. [2022]113.

## Consent

Informed consent was waived for this retrospective design.

## Sources of funding

Beijing Hospitals Authority’s Research and Translation Application of Clinical Diagnosis and Treatment Technology of the Capital (Z201100005520020).

## Author contribution

J.W. and T.W.: contributed to the preparation of the manuscript and data collection; B.Y. and Y.M.: contributed to the data collection; J.L. and J.W.: contributed to data analysis and interpretation; L.J., Y.C., P.G., Y.W., J.C., and F.C.: contributed to the experimental design and manuscript revision. All authors contributed to the article and approved the submitted version.

## Conflicts of interest disclosure

There are no conflicts of interest.

## Research registration unique identifying number (UIN)

1. Name of the registry: Impact of Acute Silent Ischemic Lesions on Clinical Outcomes of Carotid Revascularization.
2. Unique identifying number or registration ID: researchregistry9386.
3. Hyperlink to your specific registration (must be publicly accessible and will be checked): Browse the Registry-Research Registry.

## Guarantor

Liqun Jiao, Department of Neurosurgery and Department of Interventional Radiology, Xuanwu Hospital, Capital Medical University, No. 45 Changchun Street, Xicheng District, Beijing 100053, People’s Republic of China. E-mail: liqunjiao@sina.cn Yan Ma, Department of Neurosurgery, Xuanwu Hospital, Capital Medical University, No. 45 Changchun Ave, Xicheng District, Beijing 100053, Peoples Republic of China. E-mail: leavesyan@sina.com.

## Data availability statement

The data that support the findings of this study are available from the corresponding author upon reasonable request.

## Provenance and peer review

Uninvolved.

## Supplementary Material
