# Supplementary material for: Impact of acute silent ischemic lesions on clinical outcomes of carotid revascularization
Source: Int J Surg. 2023 Dec 5;110(2):974–83. doi: 10.1097/JS9.0000000000000925 (PMC10871655; doi:10.1097/JS9.0000000000000925)
Supplement: Supplementary file 2 [file js9-110-0974-s002.docx]

Table S1. In-Hospital Perioperative Outcomes Before Propensity-Score Matching in Carotid Endarterectomy

| **Outcome** | **ASIL (N=91)** | **NASIL(N=493)** | **Absolute difference (95% CI), %** | **Relative risk (95% CI)** | ***P* value** |
| --- | --- | --- | --- | --- | --- |
| **Primary outcomes** | | | | | |
| Any stroke, myocardial infarction or all-cause death | 10(10.99) | 10(2.03) | 8.96(2.42-15.51) | 5.42(2.32-12.64) | <.001^a^ |
| **Secondary outcomes** | | | | | |
| Any stroke | 8(8.79) | 9(1.83) | 6.97(1.03-12.90) | 4.81(1.91-12.15) | <.001^a^ |
| Ischemic stroke | 6(6.59) | 5(1.01) | 5.58(0.40-10.75) | 6.50(2.03-20.85) | <.001^a^ |
| Hemorrhagic stroke | 2(2.20) | 4(0.81) | 1.39(-1.73-4.50) | 2.71(0.50-14.57) | 0.24^a^ |
| All-cause death | 2(2.20) ^b^ | 3(0.61) ^c^ | 1.59(-1.50-4.68) | 3.61(0.61-21.31) | 0.37^a^ |
| Myocardial infarction | 2(2.20) | 1(0.20) | 1.99(-1.04-5.03) | 10.84(0.99-118.25) | 0.10^a^ |
| Procedure Related Complications | 6(6.59) | 12(2.43) | 4.16(-1.12-9.44) | 2.71(1.04-7.03) | 0.08^a^ |
| Pulmonary embolism | 0 | 0 | NA | NA | NA |
| Re-operation | 1(1.10) ^d^ | 3(0.61) ^d^ | 0.49(-1.76-2.74) | 1.81(0.19-17.17) | 1.00^a^ |
| Cerebral hyperperfusion syndrome | 5(5.49) | 6(1.22) | 4.28(-0.50-9.06) | 4.51(1.41-14.48) | 0.02^a^ |
| Neck hematoma | 0 | 3(0.61) | NA | NA | NA |
| Postoperative acute silent ischemic lesions | 26(28.57) | 120(24.34) | 5.11(-5.79-14.26) | 1.17(0.82-1.68) | 0.39 |

ASIL: acute silent ischemic lesions

NASIL: non-acute silent ischemic lesions

a The cells have expected counts less than 5. Chi-Square may not be a valid test. Continuity Adj. Chi-Square was performed.

b Both patients died of cardiac arrest due to postoperative acute myocardial infarction.

c Three patients died of cerebral hernia induced by intracranial hypertension due to postoperative cerebral hemorrhage.

d Acute ipsilateral carotid artery occlusion after carotid endarterectomy, emergency thrombectomy was performed.

Table S2. In-Hospital Perioperative Outcomes Before Propensity-Score Matching in Carotid Stenting

| **Outcome** | **ASIL (N=108)** | **NASIL(N=480)** | **Absolute difference (95% CI), %** | **Relative risk (95% CI)** | ***P* value** |
| --- | --- | --- | --- | --- | --- |
| **Primary outcome** | | | | | |
| Any stroke, myocardial infarction or all-cause death | 10(9.26) | 14(2.92) | 6.34(0.67-12.01) | 3.17(1.45-6.95) | 0.01^a^ |
| **Secondary outcomes** | | | | | |
| Any stroke | 10(9.26) | 14(2.92) | 6.34(0.67-12.01) | 3.17(1.45-6.95) | 0.01^a^ |
| Ischemic stroke | 7(6.48) | 9(1.88) | 4.61(-0.19-9.41) | 3.46(1.32-9.08) | 0.02^a^ |
| Hemorrhagic stroke | 3(2.78) | 7(1.46) | 1.32(-1.96-4.60) | 1.90(0.50-7.25) | 0.58^a^ |
| All-cause death | 1(0.93) ^b^ | 2(0.42) ^c^ | 0.51(-1.39-2.41) | 2.22(0.20-24.29) | 1.00^a^ |
| Myocardial infarction | 0 | 0 | NA | NA | NA |
| Procedure Related Complications | 7(6.48) | 8(1.67) | 4.81(0.03-9.60) | 3.89(1.44-10.49) | 0.01^a^ |
| Pulmonary embolism | 1(0.93) | 0 | NA | NA | NA |
| Re-operation | 0 | 1(0.21) ^d^ | NA | NA | NA |
| Cerebral hyperperfusion syndrome | 6(5.56) | 6(1.25) | 4.31(-0.13-8.74) | 4.44(1.46-13.31) | 0.01^a^ |
| Access site bleeding complication | 0 | 1(0.21) | NA | NA | NA |
| Postoperative acute silent ischemic lesions | 64(59.26) | 281(58.54) | 0.72(-9.54-10.98) | 1.01(0.85-1.20) | 0.89 |

ASIL: acute silent ischemic lesions

NASIL: non-acute silent ischemic lesions

a The cells have expected counts less than 5. Chi-Square may not be a valid test. Continuity Adj. Chi-Square was performed.

b The patient died of cerebral hernia induced by intracranial hypertension due to postoperative cerebral hemorrhage.

c Two patients died of cerebral hernia induced by intracranial hypertension due to postoperative cerebral hemorrhage.

d Acute ipsilateral carotid artery occlusion after carotid artery stenting, emergency thrombectomy was performed.

Table S3. In-Hospital Perioperative Outcomes Between Carotid Endarterectomy and Carotid Stenting in Patients with ASIL

| **Outcome** | **CEA (N=91)** | **CAS(N=108)** | **Absolute difference (95% CI), %** | **Relative risk (95% CI)** | ***P* value** |
| --- | --- | --- | --- | --- | --- |
| **Primary outcome** | | | | | |
| Any stroke, myocardial infarction or all-cause death | 10(10.99) | 10(9.26) | 1.73(-6.71-10.17) | 1.01(0.93-1.12) | 0.69 |
| **Secondary outcomes** | | | | | |
| Any stroke | 8(8.79) | 10(9.26) | -0.47(-8.45-7.52) | 0.99(0.91-1.09) | 0.91 |
| Ischemic stroke | 6(6.59) | 7(6.48) | 0.11(-6.79-7.01) | 1.00(0.93-1.08) | 0.97 |
| Hemorrhagic stroke | 2(2.20) | 3(2.78) | -0.58(-4.90-3.74) | 0.99(0.95-1.04) | 0.79 ^a^ |
| All-cause death | 2(2.20) | 1(0.93) | 1.27(-2.24-4.78) | 1.01(0.98-1.05) | 0.46 ^a^ |
| Myocardial infarction | 2(2.20) | 0 | NA | NA | NA |
| Procedure Related Complications | 6(6.59) | 7(6.48) | 0.11(-6.78-7.01) | 1.01(0.35-2.92) | 0.97 |
| Pulmonary embolism | 0 | 1(0.93) | NA | NA | NA |
| Re-operation | 1(1.10) | 0 | NA | NA | NA |
| Cerebral hyperperfusion syndrome | 5(5.49) | 6(5.56) | -0.06(-6.43-6.31) | 1.00(0.93-1.07) | 0.99 |
| Neck hematoma /Access site bleeding complication | 0 | 0 | NA | NA | NA |
| Postoperative acute silent ischemic lesions | 26(28.57) | 64(59.26) | -30.92(-44.11- -17.73) | 0.57(0.43-0.73) | <0.01 |

ASIL: acute silent ischemic lesions

CEA: carotid endarterectomy

CAS: carotid stenting

a The cells have expected counts less than 5. Chi-Square may not be a valid test. Continuity Adj. Chi-Square was performed.

Table S4. In-Hospital Perioperative Outcomes Between Carotid Endarterectomy and Carotid Stenting in Patients with NASIL

| **Outcome** | **CEA(N=493)** | **CAS(N=480)** | **Absolute difference (95% CI), %** | **Relative risk (95% CI)** | ***P* value** |
| --- | --- | --- | --- | --- | --- |
| **Primary outcome** | | | | | |
| Any stroke, myocardial infarction or all-cause death | 10(2.03) | 14(2.92) | -0.89(-2.84-1.06) | 0.99(0.97-1.01) | 0.37 |
| **Secondary outcomes** | | | | | |
| Any stroke | 9(1.83) | 14(2.92) | -1.09(-3.00-0.82) | 0.99(0.97-1.01) | 0.26 |
| Ischemic stroke | 5(1.01) | 9(1.88) | -0.86(-2.36-0.64) | 0.99(0.98-1.01) | 0.26 |
| Hemorrhagic stroke | 4(0.81) | 7(1.46) | -0.65(-1.98-0.69) | 0.99(0.98-1.01) | 0.34 |
| All-cause death | 3(0.61) | 2(0.42) | 0.19(-0.70-1.09) | 1.00(0.99-1.01) | 0.68 ^a^ |
| Myocardial infarction | 1(0.20) | 0 | NA | NA | NA |
| Procedure Related Complications | 12(2.43) | 8(1.67) | 0.77(-1.01-2.55) | 1.46(0.60-3.54) | 0.40 |
| Pulmonary embolism | 0 | 0 | NA | NA | NA |
| Re-operation | 3(0.61) | 1(0.21) | 0.40(-0.40-1.20) | 1.00(0.99-1.01) | 0.33 ^a^ |
| Cerebral hyperperfusion syndrome | 6(1.22) | 6(1.25) | -0.03(-1.42-1.35) | 1.00(0.99-1.01) | 0.96 |
| Neck hematoma /Access site bleeding complication | 3(0.61) | 1(0.21) | 0.40(-0.40-1.20) | 1.00(0.99-1.01) | 0.33 ^a^ |
| Postoperative acute silent ischemic lesions | 120(24.34) | 281(58.54) | -35.17(-41.04- -29.31) | 0.53(0.47-0.60) | <0.01 |

NASIL: non-acute silent ischemic lesions

CEA: carotid endarterectomy

CAS: carotid stenting

a The cells have expected counts less than 5. Chi-Square may not be a valid test. Continuity Adj. Chi-Square was performed.

Table S5 Mechanism of Postoperative Ischemic Stroke

|  | **CEA** | | **CAS** | |
| --- | --- | --- | --- | --- |
|  | **ASIL (N=91)** | **NASIL(N=493)** | **ASIL (N=108)** | **NASIL(N=480)** |
| Ischemic stroke | 6 | 5 | 7 | 9 |
| Perforator occlusive | 0 | 0 | 0 | 0 |
| Artery-to-artery embolism | 4 | 3 | 2 | 6 |
| Hypoperfusion | 0 | 0 | 3 | 0 |
| Mixed | 2 | 2 | 2 | 3 |

Table S6 Effect of Preoperative mRS Scores in the ASIL and NASIL groups on In-Hospital Stroke, Myocardial Infarction or Death in CEA Patients

|  | ASIL(N=91) | NASIL(N=91) |
| --- | --- | --- |
| mRS≤3 | 9/87(10.34) | 1/89(1.12) |
| mRS>3 | 1/4(25.00) | 0/2(0.00) |

Table S7 Effect of Preoperative mRS Scores in the ASIL and NASIL groups on In-Hospital Stroke, Myocardial Infarction or Death in CAS Patients

|  | ASIL(N=107) | NASIL(N=107) |
| --- | --- | --- |
| mRS≤3 | 10/102(9.80) | 2/103(1.94) |
| mRS>3 | 0/5(0.00) | 0/4(0.00) |
